# Platelet Counts and Liver Enzymes After Gastric Bypass Surgery

**DOI:** 10.1007/s11695-017-3035-5

**Published:** 2017-12-08

**Authors:** Hans-Erik Johansson, Anna Wåhlén, Erica Aldenbäck, Arvo Haenni

**Affiliations:** 1 Department of Public Health and Caring Sciences/Geriatrics, Uppsala University, Uppsala Science Park, 75185 Uppsala, Sweden; 2 Östervåla Primary Health Care Centre, Åbygränd 2, 74046 Östervåla, Sweden; 3 Department of Surgery, Falu Lasarett and Uppsala University, Uppsala, Sweden; 4 Faculty of Medicine, Uppsala University, Uppsala, Sweden

**Keywords:** Morbid obesity, Gastric bypass, Platelet counts, Gamma-glutamyltransferase, Alanine aminotransferase, C-reactive protein, Ferritin

## Abstract

**Background:**

Obesity is associated with chronic inflammation, liver steatosis and increased liver enzymes such as gamma-glutamyltransferase (GGT) and alanine aminotransferase (ALT), markers for non-alcoholic fatty liver disease (NAFLD) and liver fat content. Increased platelet counts (PCs) are a biomarker reflecting inflammation and the degree of fibrosis in NAFLD. We investigated alterations in PCs, GGT, ALT, C-reactive protein (CRP) and ferritin after Roux-en-Y gastric bypass (RYGBP).

**Methods:**

One hundred twenty-four morbidly obese non-diabetic patients were evaluated before (baseline) and 12 months after (follow-up) RYGBP.

**Results:**

Body mass index (BMI) was reduced from 43.5 kg/m^2^ (baseline) to 31.1 kg/m^2^ (follow-up), and *p* < 0.001 and weight declined from 126.2 to 89.0 kg. PCs decreased from 303 × 10^9^ to 260 × 10^9^/l, *p* < 0.001. GGT was reduced from 0.63 to 0.38 μkat/l, *p* < 0.001. ALT decreased from 0.69 to 0.59 μkat/l, *p* = 0.006. CRP was lowered from 7.3 to 5.4 mg/l *p* < 0.001 and ferritin from 106 to 84 μg/l *p* < 0.001. The alterations in PCs correlated with the changes in CRP (*r* = 0.38, *p* = 0.001), BMI (*r* = 0.25, *p* = 0.012), weight (*r* = 0.24, *p* = 0.015) and inversely correlated with ferritin (*r* = 21, *p* = 0.036).

**Conclusions:**

PCs, GGT and ALT (markers for NAFLD), and CRP and ferritin (markers for inflammation) decreased in morbidly obese after RYGBP. The decrease in PCs correlated with alterations in CRP, BMI, weight and ferritin. The lowering of liver enzymes may reflect a lowered liver fat content and decreased general inflammation.

## Introduction

Roux-en-Y gastric bypass (RYGBP) has become a frequently used procedure for obesity treatment, reducing the onset and inducing remission of type 2 diabetes mellitus (T2DM) [1, 2]. Furthermore, bariatric surgery has also been shown to reduce cardiovascular mortality and mortality in general [3, 4]. Obesity is an inflammatory condition [5, 6] and is associated with non-alcoholic fatty liver disease (NAFLD) [7, 8]. Plasma gamma-glutamyltransferase (GGT) and alanine aminotransferase (ALT) are validated surrogate markers for NAFLD and liver fat content [9, 10] as well as markers for metabolic syndrome and predictors for death [11]. Increased platelet counts (PCs) and high concentrations of circulating C-reactive protein (CRP) have been observed in conditions with chronic inflammation such as the metabolic syndrome, as well as obesity, possibly due to secondary thrombocytosis [12, 13]. However, biopsies of liver tissue have shown that increased fibrosis is linearly associated with decreased PC [14]. Platelet count has been shown to be a valuable surrogate marker predicting the severity of fibrosis in NAFLD patients [14] and could be used to predict the activity of the disease [15]. Furthermore, high PCs are also related to cardiovascular death and all-cause mortality [16]. The impact of bariatric surgery on PCs and mechanisms of action are mostly unknown. Raoux et al. have recently suggested, due to their results, that bariatric surgery has a positive impact on platelet metabolism, possibly mediated by weight loss [17]. Dallel et al. have shown a significant decrease in PCs in patients treated with RYGBP [13]. Previously, we reported in a small pilot study decreased PCs after RYGBP and biliopancreatic diversion with duodenal switch [18]. The aim of this study was to evaluate changes in liver enzymes, (GGT, ALT), CRP, ferritin and PCs in non-smoking, non-diabetic obese patients treated with RYGBP with follow-up 1 year after surgery.

## Material and Methods

### Patients

One hundred twenty-four morbidly obese patients 18 years or older, all consecutive, undergoing RYGBP surgery (90 women, 34 men), all Caucasians, non-smoking and free from established diabetes at a single outpatient obesity centre were recruited. They were investigated preoperatively (baseline) and 1 year (follow-up) after RYGBP. The study was approved by the regional ethics review board at Uppsala University.

### RYGBP Surgery Procedure

It is considered by many to be the gold standard because of its high level of effectiveness and durability. A small gastric pouch was created (2 cm × 3 cm) and the remaining stomach is excluded. The proximal jejunum was divided 30 cm distal to the ligament of Treitz, to perform a gastrojejunal anastomosis which excluded the stomach and duodenum from passage of food. The jejunal limb (Roux limb) was made 100 cm long and the small intestinal continuity was maintained by an enteroenterostomy between the Roux limb and the proximal jejunum creating Y-shaped junction where the ingested food and the gastric acid and bile are mixed [19]. All participants were given the same kind of dietary advice after surgery and were recommended to take a daily oral supplement containing vitamins and minerals (Mitt Val Kvinna®) and an intramuscular injection of 1 mg cobalamin (vitamin B_12_) every third month.

### Test Procedures

All participants underwent physical examination and blood tests for PCs, GGT, ALT, CRP, ferritin and glucose preoperatively (baseline) and at follow-up at 1 year. Blood samples were collected from each patient (following an overnight fast) and were analysed using Equalis, quality-assured routine tests at the Department of Clinical Chemistry at Falun Hospital, County of Dalarna, Sweden.

### Clinical Measurements

Weight (kg) and height (m) were measured on standardised calibrated scales and BMI (kg/m^2^) was calculated.

### Statistics

All analyses were defined a priori. Results are presented as arithmetic means, with standard deviations. Changes between different time points were analysed using paired *t* tests. Tests were two-tailed and a *p* value < 0.05 was considered significant. Statistical software JMP 5.0 for PC (SAS Corporation, Cary, TX, USA) was used.

## Results

### Baseline Data

Patient clinical characteristics at baseline, i.e. before RYGBP, and at 1 year (follow-up) are shown in Table 1. At baseline, a correlation was observed between PCs and CRP (*r* = 0.28, *p* < 0.003).

**Table 1:** Baseline characteristics and 1 year follow-up data of 124 morbidly obese patients who underwent Roux-en-Y gastric bypass surgery

	RYGBP baseline	RYGBP 1 year	*p* for difference
Gender (women/men)	90/34	–	–
Age (years)	43.2 (11.6)	–	–
Height (cm)	170.0 (8.5)	169.3(8.4)	–
Weight (kg)	126.2 (19.4)	89.0 (13.8)	< 0.001
BMI (kg/m^2^)	43.5 (6.0)	31.1 (5.4)	< 0.001
Sagittal diameter (cm)	31.9 (3.7)	22.8 (3.7)	< 0.001
Platelet counts (×10^9^/l)	303 (66)	260 (58)	< 0.001
P-GGT (μkat/l)	0.63 (0.41)	0.38 (0.39)	< 0.001
P-ALT (μkat/l)	0.69 (0.32)	0.59 (0.21)	0.006
P-CRP (mg/l)	7.3 (3.9)	5.4 (1.8)	< 0.001
P-Ferritin (μg/l)	106 (91)	84 (65)	< 0.001
P-Glucose (mmol/l)	5.9 (0.6)	5.4 (0.4)	< 0.001
HbA1_c_ (mmol/mol)	37.9 (4.3)	33.9 (3.7)	< 0.001
Haemoglobin (g/l)	142 (12.4)	137 (10.6)	< 0.001
MCV (fL)	86.7 (5.1)	89.2 (5.1)	< 0.001

Data shown are arithmetic means (± SD). Normal range: platelets 165–390 × 10^9^/l, ALT < 0.8 μkat/l, GGT < 0.8 μkat/l, ferritin 10–175 μg/l

*BMI* body mass index, *GGT* gamma-glutamyltransferase, *ALT* alanine aminotransferase, *P* plasma, *CRP* C-reactive protein, *MCV* mean corpuscular volume

### Follow-up Data at 1 Year After RYGBP

Over the 12-month period, there were significant mean changes at baseline to follow-up regarding weight, BMI, sagittal diameter, PCs, plasma concentrations of GGT, ALT and CRP, fasting blood glucose, HbA1_c_, haemoglobin (Hb), mean corpuscular volume (MCV) and ferritin (Table 1).

**Fig. 1:**
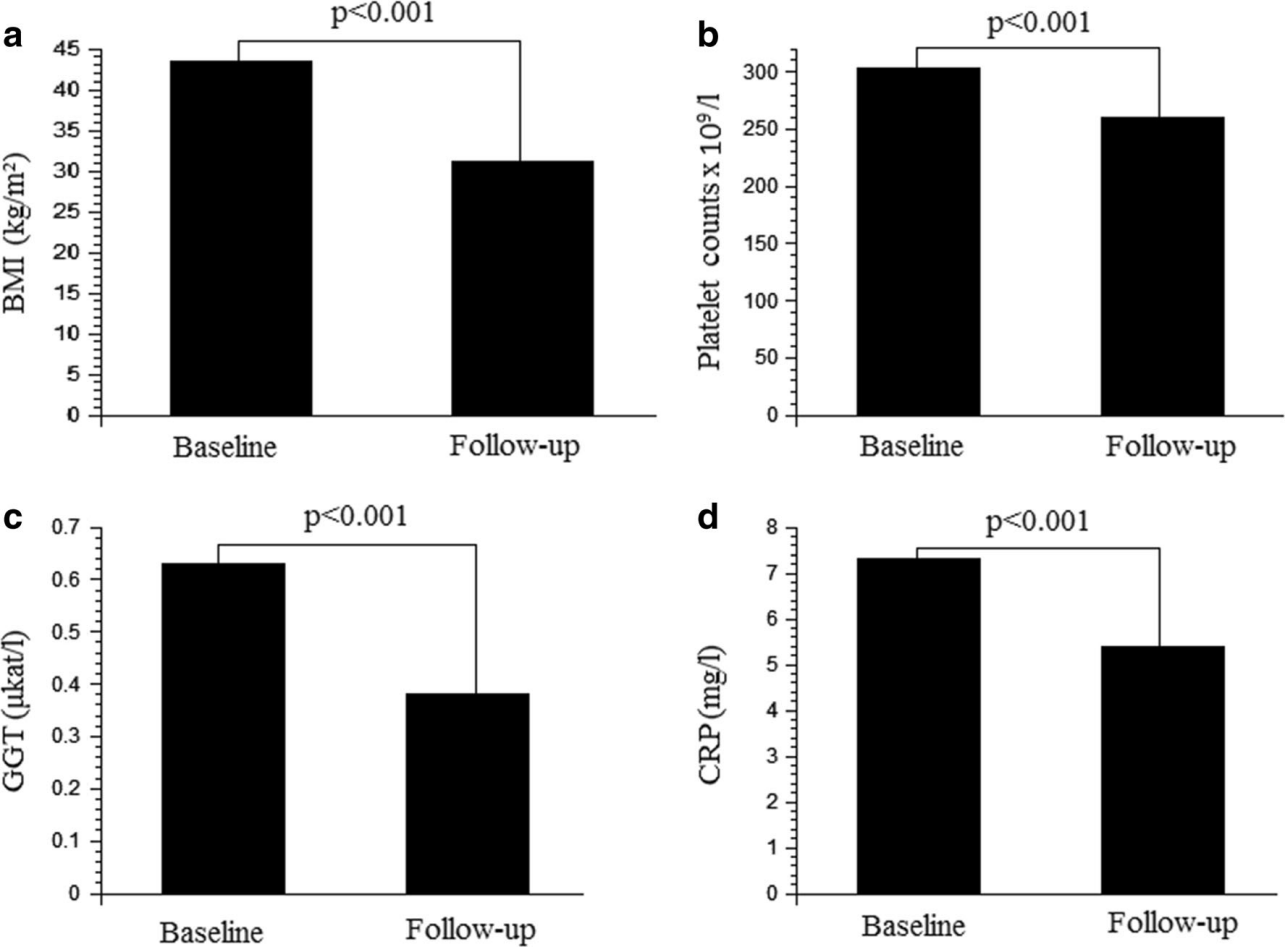
**a**–**d** The changes in body mass index (BMI) (**a**), platelet counts (**b**), concentrations of gamma-glutamyltransferase (GGT) (**c**) and C-reactive protein (CRP) (**d**) are shown at baseline, i.e. before surgery and at follow-up (1 year). Mean values are shown. Statistical significance is indicated by *p* values

Weight was lowered by 29%, from 126.2 kg at baseline to 89.0 kg at follow-up (*p* < 0.001) and sagittal diameter was reduced by 29%, from 31.9 cm at baseline to 22.8 cm at follow-up (*p* < 0.001). BMI decreased by 28%, from 43.5 kg/m^2^ at baseline to 31.1 kg/m^2^ at follow-up (*p* < 0.001), as shown in Fig. 1a.

PCs were reduced by 14%, from 303 × 10^9^/l at baseline to 260 × 10^9^/l at follow-up (*p* < 0.001) as presented in Fig. 1b.

GGT was markedly lowered by 40%, from 0.63 μkat/l at baseline to 0.38 μkat/l at follow-up (*p* = 0.011) as shown in Fig. 1c. ALT was reduced by 18%, from 0.69 μkat/l at baseline to 0.59 μkat/l at follow-up (*p* = 0.006).

CRP decreased by 25%, from 7.3 mg/l at baseline to 5.5 mg/l at follow-up (*p* < 0.001) as presented in Fig. 1d. Ferritin was lowered by 25%, from 106 μg/l at baseline to 84 μg/l at follow-up (*p* < 0.001).

The plasma fasting glucose concentration was reduced by 8%, from 5.9 mmol/l at baseline to 5.4 mmol/l at follow-up (*p* < 0.001), and HbA1_c_ was lowered by 11%, from 37.9 mmol/mol at baseline to 33.9 mmol/mol at follow-up (*p* < 0.001).

Haemoglobin was lowered over the period by 4% from 142 to 137 g/l, and MCV was increased by 3% from 86.7 to 89.2 fL (both *p* < 0.001).

### Pearson’s Product-Moment Correlation Coefficients

The alterations in PCs during the 1-year follow-up (delta-value) after RYGBP correlated with the changes in CRP (*r* = 0.38, *p* = 0.001), BMI (*r* = 0.25, *p* = 0.012), weight (*r* = 0.24, *p* = 0.015) and inversely correlated with ferritin (*r* = 21, *p* = 0.036) but did not correlate with the changes in sagittal diameter (*p* = 0.289) or glucose concentration (*p* = 0.94).

## Discussion

The main findings in this study were that the circulating concentrations of CRP and liver enzymes, GGT and ALT, decreased after RYGBP surgery along with a decrease in PCs, which may indicate a decline in the general inflammatory status and decreased liver steatosis. The impact of bariatric surgery on PCs is still unclear. A 12-month follow-up study by Raoux et al. has shown positive effects of bariatric surgery by lowered PCs still within normal range and on platelet metabolism, possible mediated by weight loss by altered platelet volume which is associated with platelet hyperactivity and cardiovascular risk [17]. Regarding gastric banding, a previous study showed a non-significant trend to lower PCs over 1 year [20]. Dallal et al. have shown a significant reduction in PCs after RYGBP with a follow-up period of 1 year [13]. From a previous, small pilot study, we reported a significant reduction in PCs for both RYGBP and biliopancreatic diversion with duodenal switch (BPD-ds) during the first year after surgery. However, the reduction was sustained only in the BPD-ds group 3 years after surgery [18]. The different responses on PCs might be explained by different procedure-related effects on general inflammation and liver steatosis.

Both CRP and PCs are not only biomarkers for inflammation, but also risk factors associated with prothrombotic states, hypercoagulability and intravascular clotting [21]. Obesity is also associated with increased concentrations of acute-phase reactants such as CRP and fibrinogen which might further explain the increased risk of thromboembolism in patients treated with bariatric surgery [22]. Furthermore, higher PCs within normal range are associated with a more severe state of atherosclerosis and worse outcome in patients with myocardial infarction and stroke [23, 24] which implies that platelet count represents a useful marker of CVD risk. Overweight and obese individuals have significantly elevated PCs as compared to normal-weight individuals [23, 24]. PCs depend on a variety of factors such as physical activity, ethnicity, age and gender. However, longitudinal studies show considerable stability of steady-state PCs and the repeatability has been shown to be very high [25].

Obesity is an inflammatory condition [26, 27] and a major risk factor for the development of NAFLD, frequently observed in obese patients [28]. The increased concentrations of circulating acute-phase proteins and proinflammatory cytokines frequently observed in inflammatory conditions are explained by their increased production by hepatocytes [29]. The hepatocyte production of acute-phase proteins is in turn influenced by the degree of liver steatosis [30, 31]. NAFLD including steatosis, commonly observed in obese patients, is associated with elevated acute-phase proteins and liver enzymes [30, 32, 33]. Lowered concentrations of liver enzymes (GGT, ALT) indicate decreased inflammation and decreased fibrosis in NAFLD hepatocytes. The circulating GGT concentration is suggested to be a major predictor for alterations in inflammation and fibrosis in NAFLD hepatocytes, the two major prognostic features in liver steatosis [10]. Another acute-phase protein ferritin, also a marker for inflammation, iron deficiency and iron stores, was evaluated in this study. Iron deficiency, low ferritin, increases PCs [34] and there is an inverse relationship between ferritin and PCs [35]. Unexpectedly, we observed a lowering of both PCs and ferritin suggesting that the latter is due to decreased degree of inflammation rather than iron deficiency [36]. Over the period, mean corpuscular volume was not lowered further indicating iron deficiency as less evident. Unfortunately, we do not have data on iron and total iron binding capacity.

Conditions with a low-grade inflammation, such as obesity, are characterised by increased PCs, although the PCs may be within normal ranges [23]. However, in more severe stages of NAFLD with fibrosis, after initially increasing PCs, a consumption of thrombocytes are observed resulting in decreased PCs [37]. Thus, the initial increase in PCs observed in liver steatosis and NAFLD may be exchanged for decreased PCs in liver fibrosis, seen in more advanced stages of NAFLD, possibly with portal hypertension and splenomegaly. Yoneda et al. have used liver biopsies to evaluate the clinical usefulness of measuring PCs for predicting the severity of liver fibrosis in 1048 patients with NAFLD [14]. They suggest platelet count to be a major biomarker for this purpose, as there is a linear association between decreased PCs and increased fibrosis in the histopathology of liver. Furthermore, Garjani A et al. have evaluated 1305 patients by abdominal ultrasonography and they conclude that platelet count in NAFLD patients can serve as an indicator of the severity of the disease and they also observed a correlation between abnormal ALT and higher PCs [15].

RYGBP surgery improves or reverses NAFLD but there is still scant data on PC changes after bariatric surgery. Our data show a sustained reduction in PCs and lowered concentrations of GGT, ALT and CRP at follow-up 1 year after RYGBP surgery possibly due to a decrease of liver inflammation and liver fat content along with alterations in cytokine state, activation of acute-phase proteins, prothrombotic and proinflammatory states. The reduction in PCs observed in this study is in accordance with 1 year data from Dallal et al. [13] but needs to be confirmed in further studies.

There are several limitations in the present study such as the small number of patients and the lack of morbidly obese controls. Liver and body fat content measured by techniques, such as dual energy X-ray absorptiometry or ultrasonography, would have been desirable to find out if any alterations in fat distributions might influence the studied variables.

## Conclusions

In conclusion, morbidly obese patients treated with RYGBP show a marked and sustained decrease in CRP, GGT and ALT. A significant reduction in PC, a marker for inflammation and fibrosis in NAFLD, was observed after 1 year, which may indicate improvement in inflammatory status generally and in particular steatohepatitis.
